# High-resolution seismic tomography of Long Beach, CA using machine learning

**DOI:** 10.1038/s41598-019-50381-z

**Published:** 2019-10-18

**Authors:** Michael J. Bianco, Peter Gerstoft, Kim B. Olsen, Fan-Chi Lin

**Affiliations:** 1 NoiseLab, University of California San Diego, La Jolla, California USA; 2 Department of Geological Sciences, San Diego State University, San Diego, California USA; 3 Department of Geology and Geophysics, University of Utah, Salt Lake City, Utah USA

**Keywords:** Geophysics, Seismology, Applied mathematics

## Abstract

We use a machine learning-based tomography method to obtain high-resolution subsurface geophysical structure in Long Beach, CA, from seismic noise recorded on a “large-N” array with 5204 geophones (~13.5 million travel times). This method, called locally sparse travel time tomography (LST) uses unsupervised machine learning to exploit the dense sampling obtained by ambient noise processing on large arrays. Dense sampling permits the LST method to learn directly from the data a dictionary of local, or small-scale, geophysical features. The features are the small scale patterns of Earth structure most relevant to the given tomographic imaging scenario. Using LST, we obtain a high-resolution 1 Hz Rayleigh wave phase speed map of Long Beach. Among the geophysical features shown in the map, the important Silverado aquifer is well isolated relative to previous surface wave tomography studies. Our results show promise for LST in obtaining detailed geophysical structure in travel time tomography studies.

## Introduction

In contrast to seismic events, ocean-atmospheric interactions provide a nearly continuous excitation of the solid Earth^1,2^. Observations of these apparently random, low amplitude seismic waves on seismic sensor arrays, often referred to as seismic “noise”, can yield rich information about geophysical properties within the region of the array^3–5^. In ambient noise tomography (ANT), this noise is cross-correlated between seismic sensors over periods of days to months to obtain travel times between sensors^6–14^. These travel times are used to perform tomography, estimating the subsurface phase speed structure for a region of interest^4,6^. Despite the fact that the number of travel times in ANT can be very large (*N*(*N* − 1)/2, with *N* the number of sensors) and the coverage of a region dense, the estimation of high-resolution phase speed structure with ANT remains a difficult problem due to many factors, e.g. irregular sensor distributions, phase ambiguities in the cross-correlations, and non-isotropic noise distributions^8^. Here we obtain high-resolution subsurface geophysical structure in Long Beach, CA using a machine learning-based tomography method, called locally sparse travel time tomography (LST)^15^. Existing ANT methods potentially oversimplify true geophysical structure by assuming phase speed models are smooth. LST improves the fidelity of ANT phase speed maps by using dictionary learning^16,17^, a form of unsupervised learning, to learn the relevant geophysical characteristics directly from seismic data. Indeed, we find that the 1 Hz Rayleigh surface wave phase speed map obtained with LST illuminates geophysical features unseen in previous surface wave tomography studies of the region. Specifically, the Silverado aquifer which supplies nearly 90% of the fresh water in Long Beach is well isolated relative to previous surface wave tomography studies. Our results show LST improve estimates of geophysical structure in travel time tomography studies.

Recently, machine learning techniques have found many useful applications in seismology^18,19^, including seismic waveform classification^20,21^, event localization^11,22^, earthquake prediction^23^, and earthquake early warning^24^. In part, the success of these methods is derived from large amounts of training and ground-truth data. In ANT however, little training data exists. LST addresses this issue by using an unsupervised machine learning method, called dictionary learning, to constrain slowness features in the tomographic image. This procedure is derived from the *adaptive* dictionary learning paradigm from image processing^17,25,26^, in which dictionaries are learned directly from corrupted measurements. In adaptive image denoising^25^, the dictionary is trained on small rectangular groups of pixels, called patches, of a noisy image. In LST, slowness dictionaries are learned from patches of a least squares-regularized inversion, and are subsequently used to reconstruct a sparsity-constrained slowness image. The dictionary is initially unknown and is learned iteratively, assuming sufficiently dense ray sampling (details in Methods). Relative to previous travel time tomography methods which enforce either smooth or discontinuous models, including conventional straight ray^27^ and eikonal tomography^8^, the sparse model in LST permits both smooth and discontinuous geophysical features. This approach is related to wavelet based methods^28^, however in LST the atoms are adapted to the slowness features in the data using dictionary learning.

## Results

**Figure 1:**
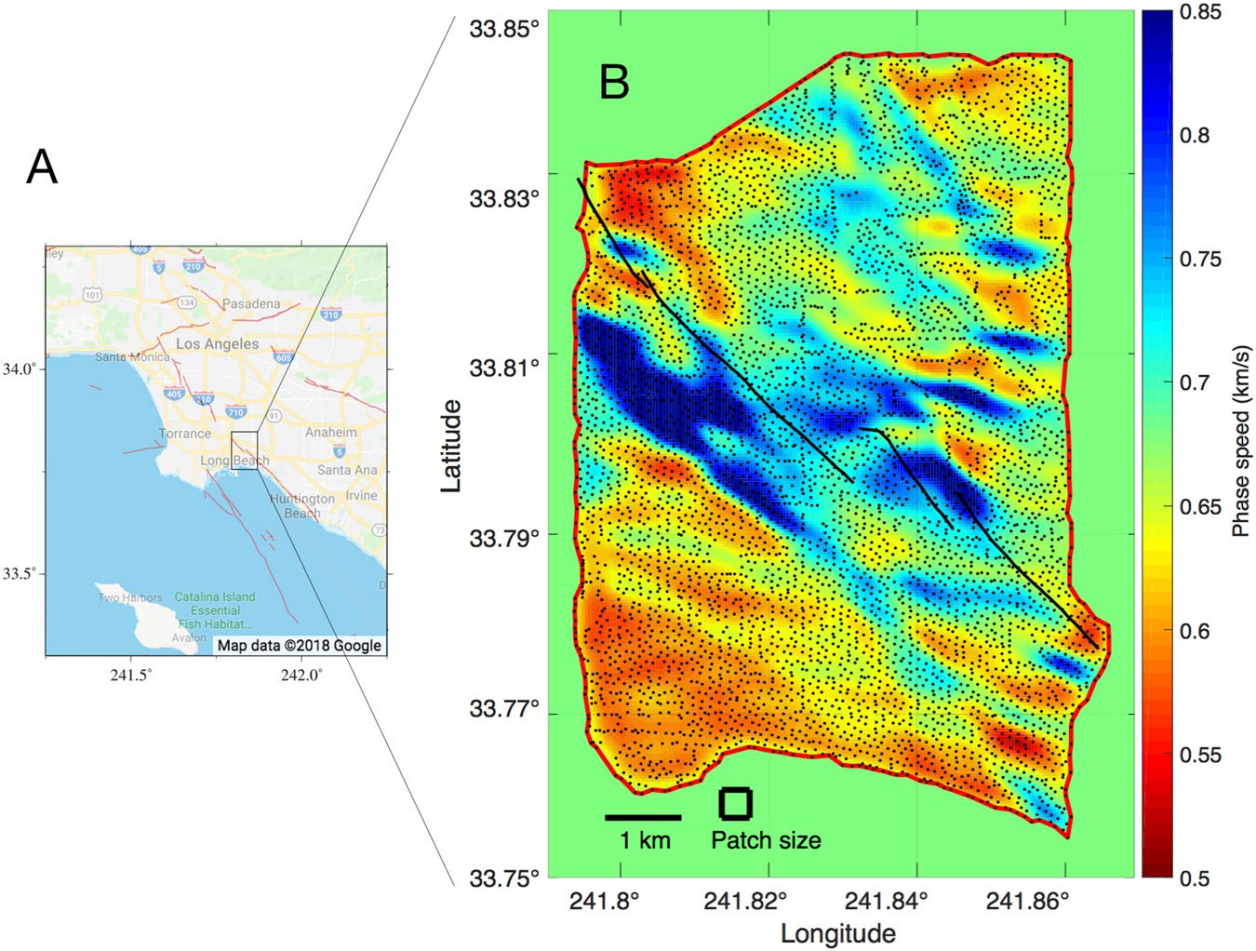
Location of Long Beach Array and LST phase speed map. The Long Beach array located in (**A**, modified from *Google Maps*^48^), contained 5204 stations (dots in (**B**)) distributed over a ~70 km^2^ area. (**B**) Locally sparse tomography (LST) phase speed maps of Long Beach, CA at 300 × 206 pixel resolution, using 3 million travel times. The Newport-Inglewood (NI) fault network (black lines) and the valid boundary for LST (red line) are shown.

Using LST, we perform surface wave ANT with data from the Long Beach array (Fig. 1A,B). The Long Beach array was deployed from January to June 2011 as part of a petroleum industry survey. It was a very dense, “large-N” array with 5204 high-frequency vertical velocity sensors distributed over a 7 × 10 km area (Fig. 1A). We obtained Rayleigh surface wave travel times between all station pairs in the array by cross-correlating seismic noise recorded during the period 5–25 March 2011. With *N* = 5204, this yielded ~13.5 million travel times, which was reduced in preprocessing. We discretized the footprint of the Long Beach array into a 300 × 206 pixel (N-S × E-W) phase-speed map (Fig. 1B), which corresponds to pixel sizes of 35 × 35 m. The phase speed for each pixel is estimated by LST with the Rayleigh wave travel times.

### Travel time data

We use the 1 Hz Rayleigh surface wave band from the Long Beach data, which corresponds to near-surface geophysical features (~100–500 m depth). For each station pair, cross-correlations of all 1-h segments from the 3-week recording were normalized and stacked to obtain the causal and anti-causal travel times. The final travel times were obtained by averaging these causal and anti-causal components^8^. After quality control the number of useful cross-correlations for 1 Hz was ~8.5 million. Quality control included SNR thresholding and removal of travel times with ranges less than one wavelength. The cross-correlations further suffered from phase ambiguities, which were reduced in preprocessing. This was done by clustering the rays^27^ and filtering travel times that exceeded the median travel times of the clusters by one half-period (0.5 s for 1 Hz). Further, cross-correlations were rejected if travel times from virtual source pairs disagreed by more than one-half period. This reduced the number of useful cross-correlations to ~3 million. For further details and preprocessing steps, see Methods and^8^.

### LST implementation

The 1 Hz Rayleigh surface wave travel times are used by LST to estimate a 300 × 206 pixel phase speed image of the Long Beach region. We assume straight-ray surface wave propagation, which yields a simple linear measurement model (Eq. 1). Using the measuments, LST alternates between solving larger-scale, or *global*, phase speed features (details in Methods) and smaller-scale or *local* phase speed features. The global problem (Eq. 4) is solved by least squares. Since the tomography matrix is sparse, we use the sparse least square program LSMR^29^. The local sparse problem (Eq. 5) is solved using orthogonal matching pursuit^25^, and the dictionary is learned using the iterative thresholding and signed K-means algorithm^30^. The reference phase speed is constant, and estimated as the average phase speed of all ray paths.

The LST tuning parameters are {*n*, *T*, *Q*, *λ*_1_, *λ*_2_}. *n* is the number of pixels per patch. We use square, 10 by 10 pixel patches, giving *n* = 100 pixels per patch (yielding 300 × 206 = 61,800 patches). We assume sparsity *T* = 2 (see Eq. 5), meaning that each patch uses two atoms from the dictionary **D**. Each atom in **D** is a vector with dimension *n* = 100 pixels (the patch size). We assume **D** has *Q* = 200 atoms, or twice the patch dimension. **D** is initialized with Gaussian random vectors of unit norm. *λ*_1_, which is the ratio of travel time error variance to global slowness variance is set as *λ*_1_ = 13 km^2^ (see Eq. 4). *λ*_2_, which is the ratio of patch slowness variance to global slowness variance, is set as *λ*_2_ = 0 assuming a sparse slowness representation (see Eq. 5). When *λ*_2_ = 0, the sparse slowness is simply the average of the patch slownesses (see Methods).

### Interpretation of phase speeds

**Figure 2:**
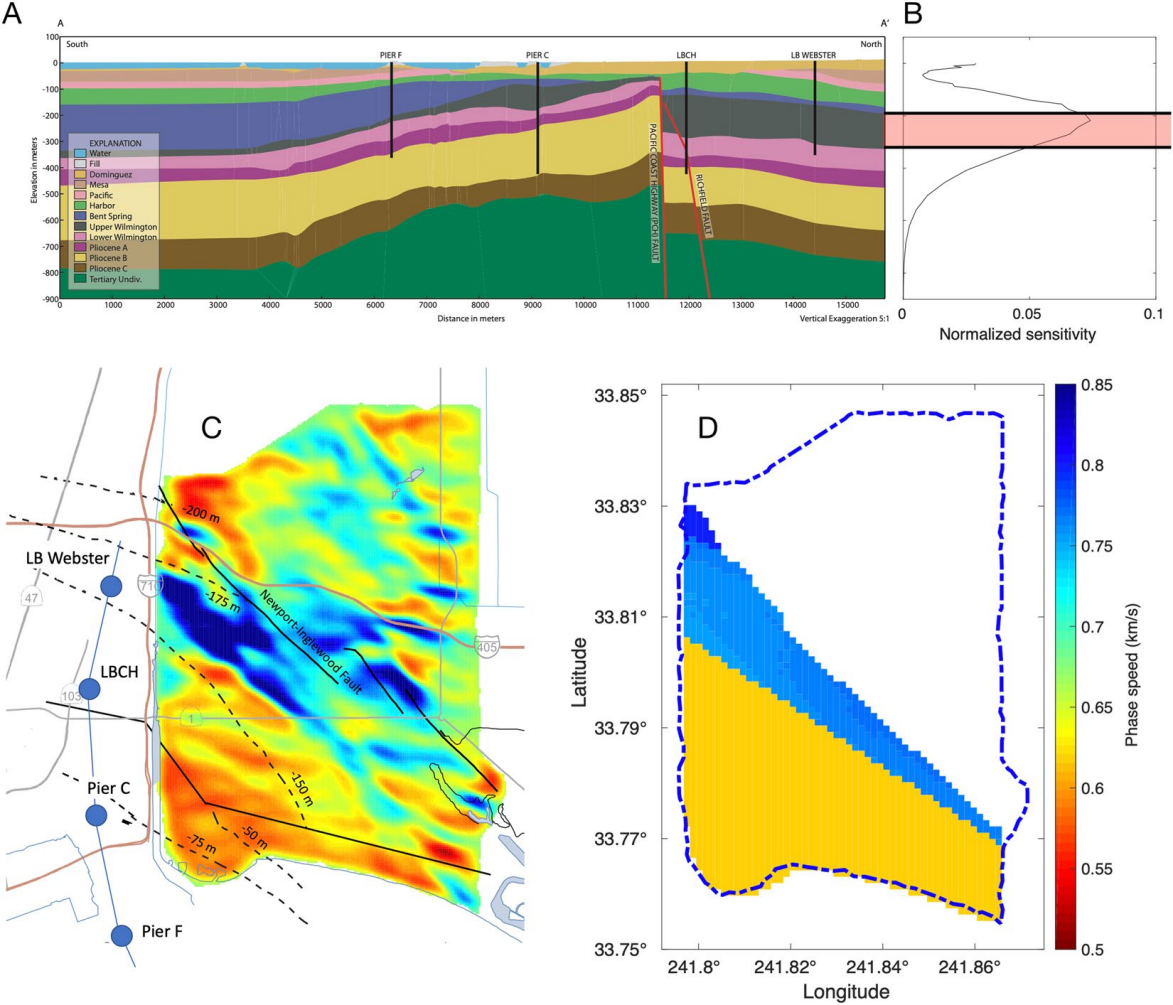
Comparison of survey-inferred geophysical features with LST phase speed map. (**A**) Inferred stratigraphy along ~N-S profile in (**C**, modified from^32^), with the 4 deep wells used as constraints. The wells are located at Pier F, Pier C, Long Beach Cabrillo High School (LBCH), and Long Beach Webster (LB Webster) elementary school, 1 km west of the Long Beach array. (**B**) 1 Hz average *V*_*s*_ depth sensitivity kernel from^8^ and overlap of northern end of the inferred Silverado (shown in (**C**)) with *V*_*s*_ sensitivity, indicated in red. (**C**) Inferred Silverado elevation^32^ overlain with LST 1 Hz Rayleigh wave phase speed map (Fig. 1B, modified from^32^), with wells (blue dots), and the location of the stratigraphy profile (blue line). (**D**) Hypothesized 1 Hz Rayleigh surface wave phase speed calculated from geological properties and inferred Silverado elevation range (**C**), from^31,32^.

In the middle of the LST phase speed image (Fig. 1B), particularly to the West, a large fast anomaly is observed between 33.78° and 33.82° latitude. This fast anomaly corresponds well with the Upper Wilmington (UW) Quaternary formation, which includes the Silverado water bearing unit (Lower Pleistocene age, ~300–580 ka) that supplies nearly 90% of the total ground water extracted in Long Beach^31,32^. Based on a 3D model^32^ (Fig. 2A) of the water-bearing structures in Long Beach obtained using borehole, seismic, and gravity surveys, the Silverado is significantly denser (2,290 kg/m^3^) than the surrounding formations (2,050–2,100 kg/m^3^) due to its coarse-grained facies. From empirical relations of density and seismic wave speeds, we expect the UW formation to increase *V*_*s*_ by ~150% relative to the surrounding formations (see Methods). Since Rayleigh wave phase speed is dependent primarily on *V*_*s*_, we conclude that this high velocity region of the map likely corresponds to the Silverado aquifer.

**Figure 3:**
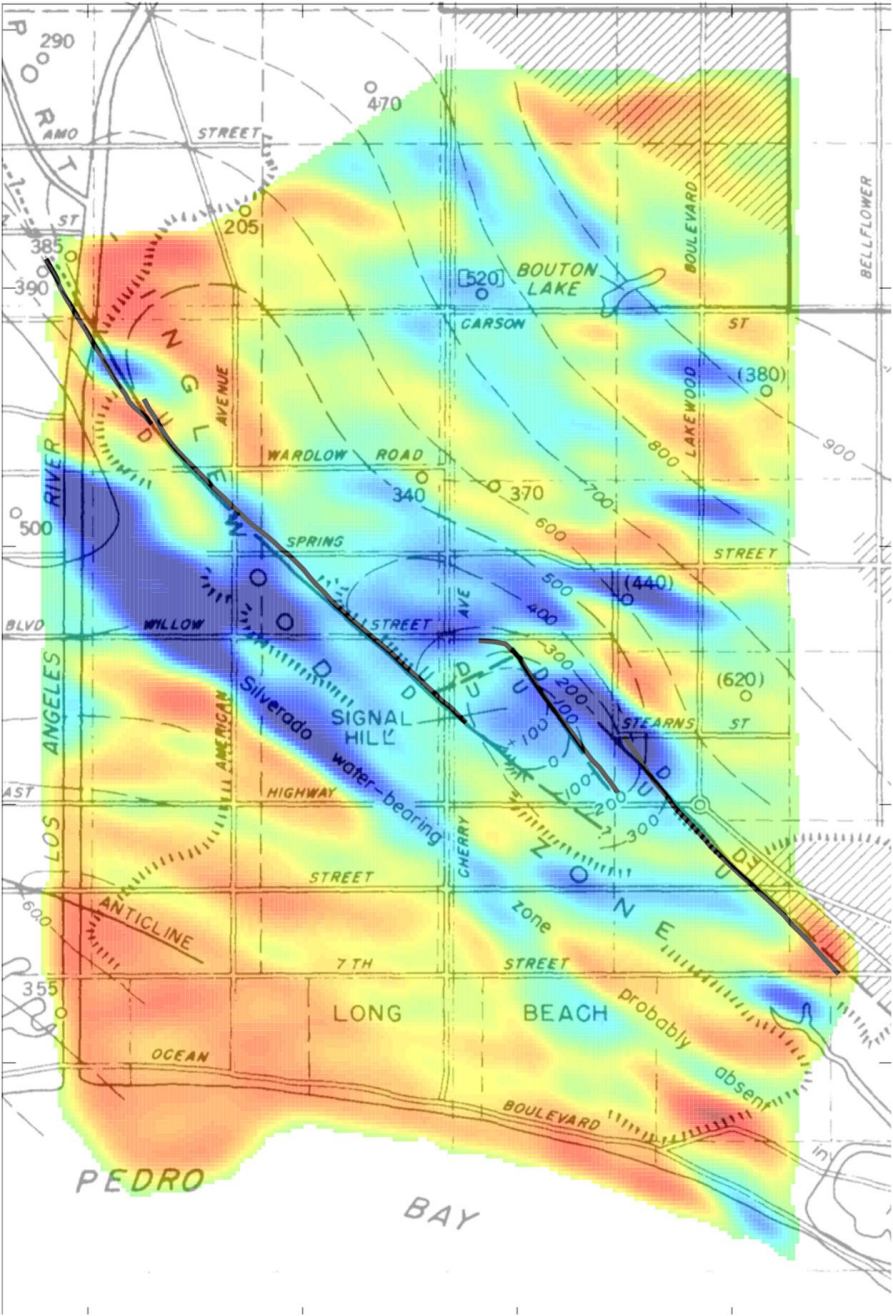
Silverado base depth (ft) inferred from borehole measurements in Poland *et al*.^31^ overlain with LST phase speed (Fig. 1B), modified from^31^. The results of^31^ contradict^32^, suggesting Silverado is absent south of the high phase speed anomaly in the west-centra part of the LST map. The findings of^31^ are corroborated by the LST result. The lower extent of the high-speed anomaly is used to generate a hypothesized phase speed map (Fig. 2D). We also note that the phase speed anomalies near and to the north of Signal Hill (indicated above) correlate well with the contours.

**Figure 4:**
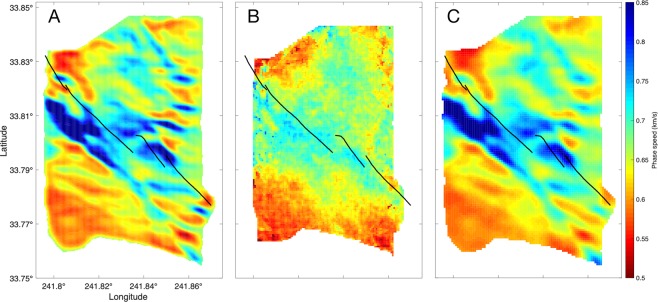
Comparison of 1 Hz Rayleigh surface wave phase speed maps from (**A**) LST, (**B**) eikonal, and (**C**) conventional tomography^8^ with the NI fault network (black lines). The general trends are the same for LST, eikonal, and conventional, though there is greater contrast and phase speed range observed in the LST map. There is greater contrast along the NI fault lines for LST. The largest disagreement between the LST/conventional result and eikonal tomography is in the western region of the map, where the LST is imaging the Silverado aquifer.

The proposed attribution of the fast anomaly to the Silverado aquifer is further supported by simulating the 1 Hz Rayleigh surface wave phase speed (Fig. 2D) using the Silverado depth range inferred in^32^ and^31^ (see Methods). In the region of the survey used from^32^, where both Silverado depth and thickness were available, the simulation shows a gradual increase in phase speed from south to north. However, per^31^, the Silverado is likely absent about 1 km south of the NI fault in the region of the Long Beach array (Fig. 3). With this assumption for the simulation, the predicted trend and magnitude and phase speeds south of the NI fault compare well with the LST result (Fig. 1B). Relative to the eikonal tomography phase speed estimates of the Long Beach (Fig. 4B), the phase speed from LST better shows the broad fast region predicted by the simulation. It is clear that the Silverado is resolved particularly well by the LST in the west-central region. Moreover, well logs support an extension of the high-velocity anomaly toward the NE across the Newport-Inglewood (NI) fault zone. This is observed in the LST phase speeds (see Fig. 2D), beyond the region of the gravity survey. The LST result appears to corroborate the older study^31^, and contradicts some of the results of^32^, which was based exclusively on a gravity survey in the region of the Long Beach array.

## Discussion

**Figure 5:**
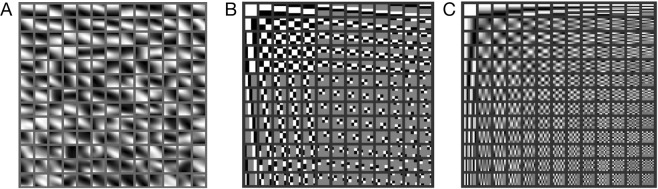
Comparison of (**A**) dictionary learned in LST inversion of Long Beach array data (Figs 1B and 2C,D), and generic dictionaries (**B**) Haar wavelet and (**C**) discrete cosine transform. All dictionaries shown with 169 atoms (*n* = 100). The atoms are sorted in order of decreasing variance from top to bottom (left to right). The learned dictionary atoms (**A**) with sharper, oriented gradients (higher variance) correspond to the sharper features in the LST phase speed map (e.g. the boundaries of the Silverado aquifer, Fig. 1B), whereas the smoother atoms (lower variance) are related to the smoother regions. Atom values stretched to full grayscale (0 to 1) for display.

These results show that the LST method, by assuming that patches of seismic phase speed fields are repetitions of a set of few patterns, contained by the dictionary, can be used to further leverage existing seismic data to obtain high-resolution phase speed images from regions of interest. Since the dictionary (Fig. 5A) is learned from the travel time data data via machine learning, it is well adapted to the true phase speeds. LST with dictionary learning provides a flexible model that is capable of modeling smooth and discontinuous slowness features. In the context of ambient noise tomography, LST leverages the dense sampling by learning the phase speed patterns. Thus, we obtain high-resolution slowness maps that well complement other geophysical sensing modalities and existing studies, for better estimating at least near-surface Earth structure. In this novel application of machine learning theory to near-surface seismic tomography, it is likely that we have further-characterized key water-bearing aquifers in Long Beach. We believe LST can help provide important geophysical insights in many tomography scenarios.

## Methods

### LST theory and implementation

Our proposed locally-sparse travel time tomography (LST) approach obtains high resolution by assuming that small patches of discrete phase speed maps are repetitions of few elemental patterns from a dictionary of patterns. Such patterns, referred to as atoms (chemistry analogy) are learned in parallel with the inversion using dictionary learning, a form of unsupervised machine learning. Relative to conventional tomography methods, the sparsity of the dictionary representation permits smooth and discontinuous, high-resolution features where warranted by the data. In the following, we present an overview of the LST theory. For more details, please see^15^.

In LST, surface wave propagation is approximated as straight ray paths through an *N* = *W*_1_ × *W*_2_ pixel phase speed map, and the travel time perturbations **t** ∈ ℝ^*M*^ from a known reference for *M* rays are modeled as

1 $${\bf{t}}={\bf{A}}{{\bf{s}}}_{{\rm{g}}}+\epsilon ,$$

where **A** ∈ ℝ^*M*×*N*^ is the tomography matrix, **s**_g_ ∈ ℝ^*N*^ is the perturbation global slowness (inverse of speed), and $\epsilon$ ∈ ℝ^*M*^ is Gaussian noise ${\mathscr{N}}(0,{\sigma }_{\varepsilon }^{2}{\bf{I}})$, with ${\sigma }_{\epsilon }^{2}$ the noise variance. We call Eq. 1 the *global model*, as it captures the large-scale features that span the discrete map and generates **t**.

We consider a second slowness model perturbation **s**_s_ ∈ ℝ^*N*^, called the sparse slowness, in which $\sqrt{n}\times \sqrt{n}$ groups of pixels are represented as sparse linear combinations of atoms from a dictionary. The patches are selected from **s**_s_ ∈ ℝ^*N*^ by the binary matrix **R** ∈ {0, 1}^*n*×*N*^, and modeled as

2 $${\hat{{\bf{x}}}}_{i}=\mathop{{\rm{\arg }}\,{\rm{\min }}}\limits_{{{\bf{x}}}_{i}}{\Vert {{\bf{R}}}_{i}{{\bf{s}}}_{{\rm{s}}}-{\bf{D}}{{\bf{x}}}_{i}\Vert }_{2}^{2}\,{\rm{subject}}\,{\rm{to}}{\Vert {{\bf{x}}}_{i}\Vert }_{0}=T,$$

where **R**_*i*_**s**_*s*_ ∈ ℝ^*n*^ is the *i*-th patch, **D** ∈ ℝ^*n*×*Q*^ is the dictionary of *Q* atoms, $\widehat{{\bf{x}}}$_*i*_ ∈ ℝ^*Q*^ coefficient estimates for the *i*-th patch, and *T* is the number of non-zero coefficients in $\widehat{{\bf{x}}}$_*i*_. We consider all overlapping patches, and wrap the patches at the edges. Thus the number of patches is *N*. The ${\ell }_{0}$ pseudo-norm penalizes the number of non-zero coefficients^25^. We call Eq. 2 the local model, as it captures the smaller scale, localized features contained by patches. The dictionary **D** is assumed unknown and is learned from the data during the inversion.

The global (Eq. 1) and local (Eq. 2) models are combined into a Bayesian *maximum a posteriori* (MAP) objective

3 $$\begin{array}{rcl}\{{\hat{{\bf{s}}}}_{{\rm{g}}},{\hat{{\bf{s}}}}_{{\rm{s}}},\hat{{\bf{X}}}\} & = & \mathop{{\rm{\arg }}\,{\rm{\min }}}\limits_{{{\bf{s}}}_{{\rm{g}}},{{\bf{s}}}_{{\rm{s}}},{\bf{X}}}\{\frac{1}{{\sigma }_{\varepsilon }^{2}}{\Vert {\bf{t}}-{\bf{A}}{{\bf{s}}}_{{\rm{g}}}\Vert }_{2}^{2}+\frac{1}{{\sigma }_{{\rm{g}}}^{2}}{\Vert {{\bf{s}}}_{{\rm{g}}}-{{\bf{s}}}_{{\rm{s}}}\Vert }_{2}^{2}+\frac{1}{{\sigma }_{p,i}^{2}}\sum _{i}\,{\Vert {\bf{D}}{{\bf{x}}}_{i}-{{\bf{R}}}_{i}{{\bf{s}}}_{{\rm{s}}}\Vert }_{2}^{2}\}\\  &  & {\rm{subject}}\,{\rm{to}}{\Vert {{\bf{x}}}_{i}\Vert }_{0}=T\,\forall \,i,\end{array}$$

where ${\widehat{{\bf{s}}}}_{{\rm{g}}}$ is an estimate of the global slowness perturbation, *σ*_g_^2^ is the global slowness variance, ${\widehat{{\bf{s}}}}_{{\rm{s}}}$ is the estimate of the sparse slowness perturbation, *σ*_*p*,*i*_^2^ is the variance of the patch slowness, and $\widehat{{\bf{X}}}$ ∈ ℝ^*Q*×*I*^ is the coefficient estimates.

We find the MAP estimates {${\widehat{{\bf{s}}}}_{{\rm{g}}}$,${\widehat{{\bf{s}}}}_{{\rm{s}}}$,$\widehat{{\bf{X}}}$} using a block-coordinate minimization algorithm by decoupling the local and global models via substitution^17,25^. The global objective is, from Eq. 3,

4 $${\hat{{\bf{s}}}}_{{\rm{g}}}=\mathop{{\rm{\arg }}\,{\rm{\min }}}\limits_{{{\bf{s}}}_{{\rm{g}}}}{\Vert {\bf{t}}-{\bf{A}}{{\bf{s}}}_{{\rm{g}}}\Vert }_{2}^{2}+{\lambda }_{1}{\Vert {{\bf{s}}}_{{\rm{g}}}-{{\bf{s}}}_{{\rm{s}}}\Vert }_{2}^{2},$$

where *λ*_1_ = (*σ*_ε_/*σ*_g_)^2^ is a regularization parameter. The local objective is from Eq. 3, substituting **s**_s_ = ${\widehat{{\bf{s}}}}_{{\rm{g}}}$,

5 $${\hat{{\bf{x}}}}_{i}=\mathop{{\rm{\arg }}\,{\rm{\min }}}\limits_{{{\bf{x}}}_{i}}{\Vert {\bf{D}}{{\bf{x}}}_{i}-{{\bf{R}}}_{i}{\hat{{\bf{s}}}}_{{\rm{g}}}\Vert }_{2}^{2}\,{\rm{subject}}\,{\rm{to}}{\Vert {{\bf{x}}}_{i}\Vert }_{0}=T.$$

Dictionary learning is added to the local problem (5), by optimizing **D**:

6 $$\hat{{\bf{D}}}=\mathop{{\rm{\arg }}\,{\rm{\min }}}\limits_{{\bf{D}}}\{\mathop{{\rm{\min }}}\limits_{{{\bf{x}}}_{i}}{\Vert {\bf{D}}{{\bf{x}}}_{i}-{{\bf{R}}}_{i}{\hat{{\bf{s}}}}_{{\rm{g}}}\Vert }_{2}^{2}\,{\rm{subject}}\,{\rm{to}}{\Vert {{\bf{x}}}_{i}\Vert }_{0}=T\,\forall \,i\}.$$

The dictionary learning problem (Eq. 6) is here solved using the iterative thresholding and signed k-means (ITKM) algorithm^30^. After $\widehat{{\bf{D}}}$ is obtained, the coefficients $\widehat{{\bf{X}}}$ = [$\widehat{{\bf{x}}}$_1_, ..., $\widehat{{\bf{x}}}$_*I*_] are solved from Eq. 5 using orthogonal matching pursuit (OMP) with the same sparsity level *T* as ITKM. Then with $\widehat{{\bf{X}}}$, $\widehat{{\bf{D}}}$, and global slowness ${\widehat{{\bf{s}}}}_{{\rm{g}}}$ from Eq. 4 we solve for **s**_s_. Equation 3 gives, assuming constant patch variance *σ*_*p*,*i*_^2^ = *σ*_*p*_^2^,

7 $${\hat{{\bf{s}}}}_{{\rm{s}}}=\mathop{{\rm{\arg }}\,{\rm{\min }}}\limits_{{{\bf{s}}}_{s}}\,{\lambda }_{2}{\Vert {\hat{{\bf{s}}}}_{{\rm{g}}}-{{\bf{s}}}_{{\rm{s}}}\Vert }_{2}^{2}+\sum _{i}\,{\Vert {\bf{D}}{\hat{{\bf{x}}}}_{i}-{{\bf{R}}}_{i}{{\bf{s}}}_{{\rm{s}}}\Vert }_{2}^{2},$$

where *λ*_2_ = (*σ*_*p*_/*σ*_g_)^2^ is a regularization parameter. The solution to Eq. 7 is analytic

8 $${\hat{{\bf{s}}}}_{{\rm{s}}}=\frac{{\lambda }_{2}{\hat{{\bf{s}}}}_{{\rm{g}}}+n{{\bf{s}}}_{p}}{{\lambda }_{2}+n},$$

where *n* is the number of patches and ${{\bf{s}}}_{p}=\frac{1}{n}\sum _{i}\,{{\bf{R}}}_{i}^{{\rm{T}}}{\bf{D}}{\hat{{\bf{x}}}}_{i}$. Equation 8 gives **s**_s_ as the weighted average of the patch slownesses {**D**$\widehat{{\bf{x}}}$_*i*_∀*i*} and ${\widehat{{\bf{s}}}}_{{\rm{g}}}$. When *λ*_2_ ≪ *n*, **s**_s_ ≈ **s**_*p*_. When *λ*_2_ = *n*, **s**_g_ and **s**_*p*_ have equal weight. It is typical in image denoising to set *λ*_2_ = 0^26^. The expressions Eq. 4–8 are solved iteratively until convergence. Before solving Eq. 5 and 6, the slowness patches {**R**_*i*_${\widehat{{\bf{s}}}}_{{\rm{g}}}$∀*i*} are centered^26^, i.e. the mean of the pixels in each patch is subtracted. The mean of patch *i* is ${\bar{x}}_{i}=\frac{1}{n}{1}^{{\rm{T}}}{{\bf{R}}}_{i}{\hat{{\bf{s}}}}_{{\rm{g}}}$. Hence, **R**_*i*_${\widehat{{\bf{s}}}}_{{\rm{g}}}$ ≈ **Dx**_*i*_ + 1*x*_*i*_. The tomographic image used for geophysical interpretation from the LST algorithm is ${\widehat{{\bf{s}}}}_{{\rm{s}}}$.

**Figure 6:**
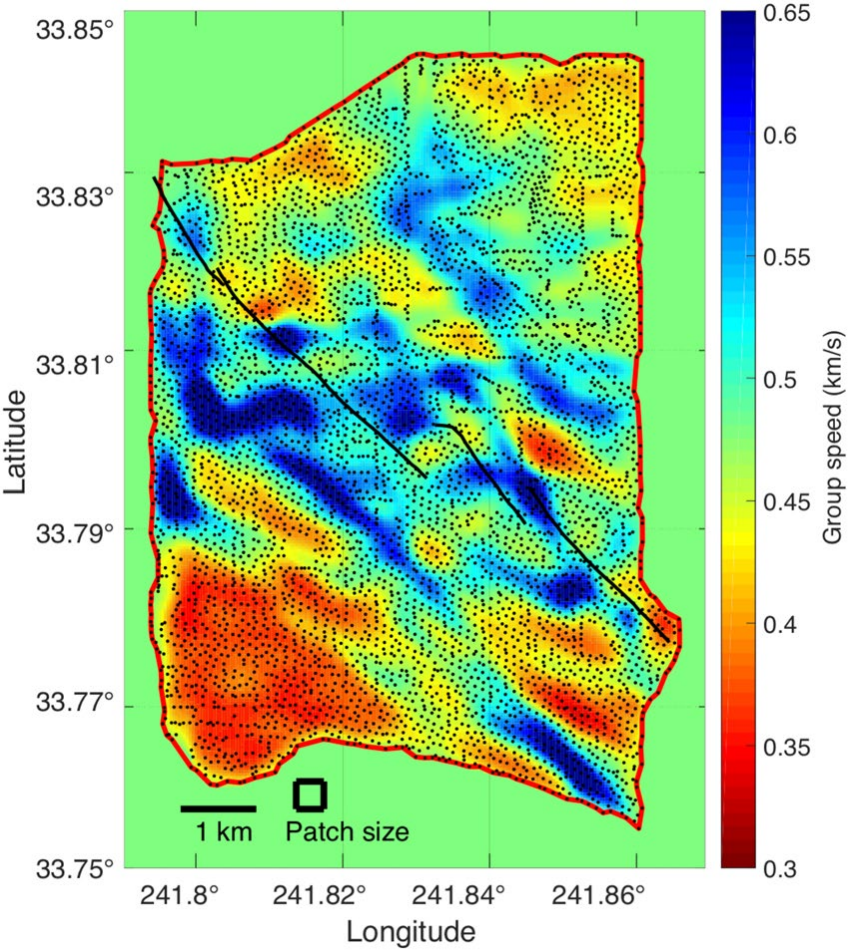
1 Hz Rayleigh surface wave group speed map from LST, estimated using the same inversion parameters as the LST phase speed map (Fig. 4A). The group speed map shares the same speed trends as the LST phase speed map, including the large fast anomaly south of the NI fault corresponding to the Silverado aquifer. The stations (dots) and the valid boundary for LST (red line) are shown.

We note that the LST approach performs well for both prescribed (e.g. wavelet and DCT) and learned dictionaries (see Fig. 5). But in general we expect the tomographic image fidelity of the learned dictionary inversion to be greater than that of prescribed dictionaries. This follows the results of simulations in^15^ (e.g. Figures 5, 6, 8 and 9), which compares the performance of prescribed and wavelet dictionaries on synthetic slowness maps and shows learned dictionaries can reduce the RMSE relative to ground truth by greater than 50% over prescribed dictionaries. This also follows the “synthesis” (vs. “analysis”) paradigm in image processing, which prefers to learn dictionaries if sufficient training data is available rather than use e.g. wavelet functions which must be justified by detailed theoretical analysis for each application. Dictionaries can be learned from a corpus of data, or as was done here, from a large number of examples from one imaging scenario. This has proven a successful approach in e.g. image restoration^16^ and medical imaging^17^. For more details please see^25^ (pp. 227–246).

### Conventional tomography

We implement conventional tomography using a Bayesian approach^33^, which regularizes the inversion with a global smoothing (non-diagonal) covariance. Considering the measurements (1), the MAP estimate of the slowness is

9 $${\hat{{\bf{s}}}}_{{\rm{g}}}={({{\bf{A}}}^{{\rm{T}}}{\bf{A}}+{{\rm{\eta }}\Sigma }_{{\rm{L}}}^{-1})}^{-1}{{\bf{A}}}^{{\rm{T}}}{\bf{t}},$$

where *η* = (*σ*_*ε*_/*σ*_*c*_)^2^ is a regularization parameter, with *σ*_c_ the conventional slowness variance, and

10 $${\Sigma }_{{\rm{L}}}(i,j)=\exp (-{l}_{i,j}/L).$$

Here *l*_*i*,*j*_ is the distance between cells *i* and *j*, and *L* is the smoothness length scale^33,34^. For our simulation we choose *L* = 10 km and *η* = 100 km^2^. This gives the best tradeoff between detail and fidelity.

### LST, eikonal, and conventional performance comparisons

We quantify the relative quality of the LST and eikonal phase speed maps (Fig. 4) by variance reduction, and using visual quality scores derived from natural images. The LST provides a 57% variance reduction and conventional gives 59%, whereas eikonal gives a 28% reduction. The increased variance reduction in the LST and conventional inversions over the eikonal result is likely because the eikonal tomography method does not explicitly minimize the travel time residual. Both LST and conventional tomography minimize the travel time residual in their map estimation (Eq. 4 and 9). Since there is no true phase speed map available, we use reference-less image quality metrics to help quantify the quality of the LST and eikonal phase speeds. While such metrics may not reflect the truth of estimated geophysical features, they can help quantify corruption of the geophysical features. We use the Blind/Referenceless Image Spatial Quality Evaluator (BRISQUE)^35^, the Natural Image Quality Evaluator (NIQE)^36^, and the Perception based Image Quality Evaluator (PIQE)^37^. The results are summarized in Table 1. Overall, LST obtains a better score on 2 of 3 of the metrics. The BRISQUE metric has incorporated human opinions of image quality, whereas NIQE and PIQE do not. Hence BRISQUE may be less suited to our application.

**Table 1:** Reference-less image quality score for Conventional, Eikonal, and LST methods with BRISQUE, NIQE, and PIQE metrics (lower score is better).

Method	BRISQUE	NIQE	PIQE
Conventional	51.2	10.1	88.4
Eikonal	**43.7**	11.0	86.8
LST	45.8	**5.9**	**72.5**

Since surface wave phase travel times potentially suffer from phase ambiguities, we also performed an LST inversion of the 1 Hz Rayleigh wave group travel times using the same parameters as the LST phase speed map (Fig. 4A). The LST group speed map is shown in Fig. 6. The group travel times are not subject to phase ambiguities, but may present other problems such as erratic arrival picks. The trends in LST group speed map (Fig. 6) compare well with those of the LST phase speed map (Fig. 4A), and both show a large fast anomaly south of the NI fault. We note that the group speed is slower overall, than the phase speed map, which is expected.

We compare the results of the LST to eikonal tomography^8,38^ and conventional tomography (Fig. 4). Eikonal tomography avoids the inversion of large tomography matrices in favor of solving a number of simpler subproblems, while also partly accounting for wave refraction. For each virtual source, a phase speed map is estimated from the local gradients of the smoothed travel time surface. The individual phase speed estimates from all the virtual sources are then averaged to obtain the final phase speed map. Since LST uses the straight ray assumption, it is more similar to conventional than eikonal tomography.

However, LST provides improved results over conventional tomography with less computational burden. Conventional tomography^33^ complexity is dominated by square matrix inversion ${\mathscr{O}}({N}^{3})$, though approximate solution methods are slightly less complex. For large tomography matrices **A**, LST is dominated by matrix multiplication in the LSMR algorithm^29^ ${\mathscr{O}}(2MN)$. Since for LST the sparse matrix **A** is used directly, the memory required for a tomography problem with *M* travel times scales linearly with the map size *N*. For conventional tomography, the memory required scales by *N*^2^. Hence LST could be used for much larger maps than conventional tomography.

### Geological interpretation

The LST sensitivity depth range (~100–500 m) with 1 Hz Rayleigh surface waves is occupied by Pleistocene and Holocene deposits^32^, which contain almost all the ground water resources in the area, Fig. 3A–C. The Silverado formation (Lower Pleistocene age, ~300–580 ka, named in^31^), accounts for nearly 90% of the total ground water extraction in the region considered here^39^. The producing aquifers consist of sand and gravel, and in particular the Silverado unit is characterized by coarse-grained sediments.

Ponti *et al*.^32^ generated a 3D sequence stratigraphy model of Quaternary layers at the Dominguez Gap in a 16.5 × 16.1 km area that overlaps with our LST model region (see Fig. 2). The study was aided by 5 reference boreholes drilled to more than 450 m depth. In addition, more than 300 oil and water wells were compiled and used in the study. An important finding from this study was a fault, striking W-NW in agreement with the general trend of the area, named the Pacific Coast Highway (PCH) fault, with a progressive vertical throw (up to 200 m) causing displacement of all pre-Holocene formations down to the north (see Fig. 2).

An area-wide gravity survey^32^ was carried out to invert for stratigraphy along a N-S profile, ~1 km west of the LST result region (see Fig. 2). The Silverado aquifer has significantly higher density (2290 kg/m^3^), due to its coarse-grained facies, than the surrounding formations (2050–2100 kg/m^3^). The Silverado gravity anomaly is in general agreement with the sequence-stratigraphic model, including the termination of the Silverado unit just south of LBCH. However, the Poland *et al*.^31^ survey contradicts the Ponti *et al*.^32^ inferred stratigraphy. In^31^, it is concluded that the Silverado is missing about 1 km south of the NI fault - near the termination of the LST phase speed anomaly (see Fig. 3).

Several established empirical relations positively correlate seismic wave speeds with density. Gardner *et al*.^40^ relates density *ρ* to P-wave speed *V*_*p*_ as

11 $$\rho =0.31{V}_{p}^{0.25},$$

where *V*_*p*_ is in m/s. Equation 11 gives a 40–50% increase in *V*_*p*_ for the Silverado formation, over the surrounding Pliocene, Pleistocene and Holocene layers. Brocher^41^ related *V*_*p*_, *V*_*s*_ by

12 $${V}_{s}=0.7858-1.2344{V}_{p}+0.7949{V}_{p}^{2}-0.1238{V}_{p}^{3}+0.0064{V}_{p}^{4},$$

where *V*_*s*_ is in km/s. Equation 12 gives a 150% increase in *V*_*s*_ using derived values of *V*_*p*_^40^ for the densities of the Silverado and surrounding layers. Since Rayleigh surface wave phase speed is dependent primarily on *V*_*s*_^42^, these results suggest that the Silverado aquifer should give rise to a significant phase-speed anomaly.

**Figure 7:**
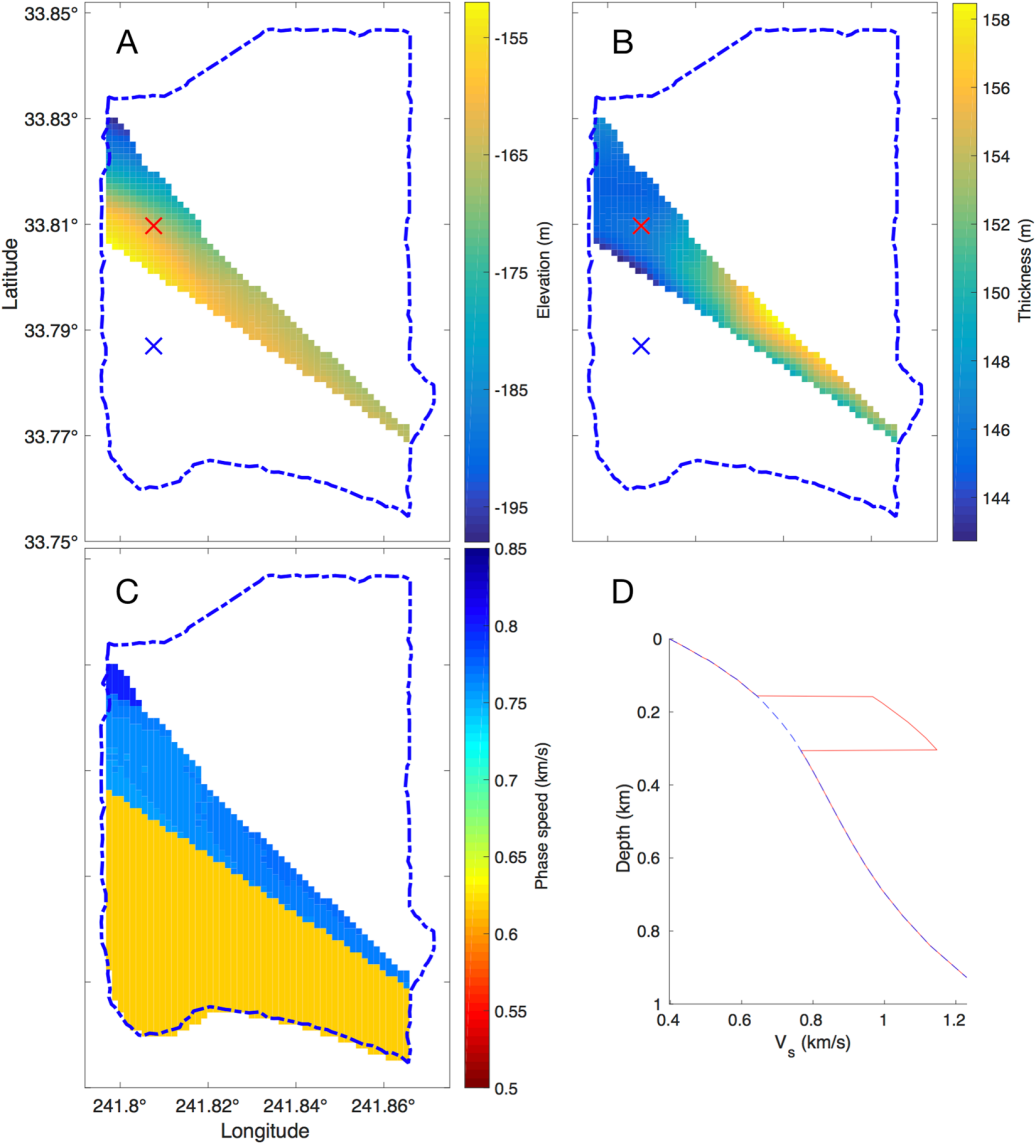
Silverado depth range and phase speed predictions using Ponti *et al*. (2007) and Poland *et al*. (1956) surveys. (**A**) Silverado elevation and (**B**) thickness from the Ponti *et al*. ^32^ survey. Silverado is missing south of LST high speed anomaly per Poland *et al*.^31^ survey (see Fig. 3). (**C**) Phase speed predicted from (A,B) with Silverado phase speed perturbation (150% of average *V*_*s*_ from^8^ from Eq. 11 and 12). (**D**) Two *V*_*s*_ profiles: estimated *V*_*s*_ with Silverado layer (red line, from location of red ‘x’ in (**A**,**B**)) and *V*_*s*_ without Silverado (mean *V*_*s*_ from^8^ (blue dashed line, from location of blue ‘x’ in (**A**,**B**)). The valid boundary for LST inversion shown as blue dashed line.

To help verify the fast anomaly in the LST result (Fig. 1B), we calculate the 1 Hz Rayleigh surface wave phase speed for the survey region based on predicted Silverado *V*_*s*_ from empirical relations Eq. 11 and 12. The method and results are summarized in Fig. 7. The Silverado elevation and thickness inferred in^32^ are interpolated (Fig. 7A,B) to obtain the depth ranges of the aquifer within the survey region. It is assumed per^31^ that Silverado is missing south of the fast anomaly. Further, we assume an average *V*_*s*_ profile for the region based on Rayleigh wave dispersion measurements in^8^. For each discrete Silverado depth range, the Silverado *V*_*s*_ is simulated by doubling the average *V*_*s*_ in the Silverado depth range (e.g. Fig. 7D). The 1 Hz Rayleigh surface wave phase speed for the survey region (Fig. 7C) is estimated using numerical forward modeling^43^.

We also consider the effect of fluid saturation of the local strata in qualifying the high-velocity anomaly in our 1 Hz phase map. The depth to the water table in Long Beach (average from 150 wells^44^) is about 7 m. Below the water table, the layers may be considered ‘wet’ to the largest depths in the deep water wells from^32^ (Pliocene age, 450 m+), though the layers are not necessarily ‘water-bearing’ per ground water extraction terminology. While laboratory experiments have shown for large *V*_*s*_ that the presence of fluids tends to decrease *V*_*s*_ of rock, this trend is insignificant for *V*_*s*_ ≈ 1,000 m/s^45^. The phase speeds observed in the high-velocity anomaly detected in our study are within this range. Thus, we conclude it unlikely that the high-velocity anomaly in our 1 Hz phase map is caused by a contrast in wet versus dry rock.

Other parameters, such as confining pressure and anisotropy^46^, as well as temperature^47^, may affect seismic velocities of the individual strata. However, we do not have measurements of these parameters available for the layers of our model area. Thus, to the best of our knowledge based on recent literature, the high-velocity anomaly in the west-central part of the Long Beach model is caused by the higher-density gravel/coarse sand of the lower Pleistocene Silverado aquifer, as supported by gravity inversion and borehole logs^32^.
